# Corticospinal activation confounds cerebellar effects of posterior fossa stimuli

**DOI:** 10.1016/j.clinph.2009.08.021

**Published:** 2009-12

**Authors:** Karen M. Fisher, H. Ming Lai, Mark R. Baker, Stuart N. Baker

**Affiliations:** a Department of Neurophysiology, Royal Victoria Infirmary, Queen Victoria Road, Newcastle-upon-Tyne NE1 4LP, UK; b Institute of Neuroscience, Henry Wellcome Building, Medical School, Newcastle University, Newcastle-upon-Tyne NE2 4HH, UK

**Keywords:** Cerebellar, Corticospinal, Motor cortex, Posterior fossa, Transcranial magnetic stimulation

## Abstract

**Objective:**

To investigate the efficacy of magnetic stimulation over the posterior fossa (PF) as a non-invasive assessment of cerebellar function in man.

**Methods:**

We replicated a previously reported conditioning-test paradigm in 11 healthy subjects. Transcranial magnetic stimulation (TMS) at varying intensities was applied to the PF and motor cortex with a 3, 5 or 7 ms interstimulus interval (ISI), chosen randomly for each trial. Surface electromyogram (EMG) activity was recorded from two intrinsic hand muscles and two forearm muscles. Responses were averaged and rectified, and MEP amplitudes were compared to assess whether suppression of the motor output occurred as a result of the PF conditioning pulse.

**Results:**

Cortical MEPs were suppressed following conditioning-test ISIs of 5 or 7 ms. No suppression occurred with an ISI of 3 ms. PF stimuli alone also produced EMG responses, suggesting direct activation of the corticospinal tract (CST).

**Conclusions:**

CST collaterals are known to contact cortical inhibitory interneurones; antidromic CST activation could therefore contribute to the observed suppression of cortical MEPs.

**Significance:**

PF stimulation probably activates multiple pathways; even at low intensities it should not be regarded as a selective assessment of cerebellar function unless stringent controls can confirm the absence of confounding activity in other pathways.

## 1 Introduction

Many clinical conditions compromise the cerebellum, and an objective way of assessing cerebellar function would be invaluable for diagnosis and in monitoring disease progression. However, non-invasive electrophysiological study of the cerebellum is difficult because of its deep location. One promising approach is to deliver electrical (Ugawa et al., 1991) or magnetic (Ugawa et al., 1995) stimulation over the posterior fossa (PF). This can suppress a motor evoked potential (MEP) elicited by motor cortical stimulation 5–7 ms later. Although PF stimulation could activate multiple structures, evidence to date suggests that the most important is a cerebellothalamocortical pathway. MEP suppression is absent in patients with cerebellar lesions (Ugawa et al., 1994a, 1995, 1997; Matsunaga et al., 2001), but normal in ataxia of non-cerebellar origin.

Despite the potential for using this technique as a diagnostic tool, concern remains that PF stimulation does not specifically activate the cerebellum. Activation of neck muscles and their afferents could provide a sensory volley which might modulate cortical excitability. Current spread into the brainstem may additionally activate ascending and descending pathways such as the corticospinal tract (CST). An antidromic CST volley could suppress motor cortical cells via recurrent collaterals (Renaud and Kelly, 1974).

In this study, we assessed cortical suppression following PF stimulation at five different intensities, and correlated this with the occurrence of direct CST activation. These two effects often occurred in parallel, leading us to conclude that PF stimulation is unlikely to be a specific tool for cerebellar assessment.

## 2 Methods

Eleven healthy volunteers (age 23–54 years, 10 males and 1 female) consented to participate in this study, which was approved by the Local Research Ethics Committee.

Subjects sat resting their head on a custom-made frame, modelled on an ophthalmic slit lamp. Straps immobilised the head with the neck flexed. Magnetic stimulation coils were clamped rigidly to the frame.

Surface electromyogram (EMG) activity was recorded (bandpass 30 Hz to 2 kHz), from the right first dorsal interosseous (1DI), abductor pollicis brevis (AbPB), extensor digitorum communis (EDC) and flexor digitorum superficialis (FDS) muscles. When a steady contraction was required, subjects squeezed the levers of an auxotonic precision grip manipulandum (Riddle and Baker, 2005) between finger and thumb. Lever displacement had to exceed 17 mm; below this, a tone sounded until the criterion displacement was achieved.

Motor cortical stimulation used a Magstim 200 stimulator and circular coil over the vertex, with current direction optimal for left hemisphere activation. Stimulus intensity was set to generate an MEP from 1DI/AbPB at approximately 0.5 mV at rest (MEP sizes: 1DI, 0.53 ± 0.09 mV; AbPB, 0.27 ± 0.07 mV; EDC, 0.17 ± 0.03 mV; FDS, 0.21 ± 0.04 mV, mean ± SEM across subjects). PF stimulation used a double cone coil and another Magstim 200. We first identified the threshold intensity for direct activation of CST fibres with the coil centred over the inion, oriented to produce a downward current in the brain. This was termed the direct motor threshold (DMT). Subjects produced a gentle background contraction, and stimulus-triggered EMG from 1DI and AbPB were viewed on an oscilloscope. Stimulus intensity was increased in 1% increments until a response above background was observed. Threshold was defined as the intensity of stimulation which produced an MEP in 50% of stimuli. MEP latencies were a few milliseconds shorter than responses to motor cortical stimuli, consistent with brainstem CST activation. Mean threshold intensity was 75% maximum stimulator output (MSO; range: 60–100%). The coil was then repositioned midway between the inion and the right mastoid incisura. Intensities used during the experiment ranged from DMT-20% to DMT, in 5% steps.

Two experiments were carried out. In the first, subjects made a background contraction and PF stimuli (0.2 Hz) were given at a randomly chosen intensity. The coil remained oriented to produce a downward brain current since we found this to be the most sensitive method of detecting CST activation in our subjects. This experiment was performed with the muscle active to produce tonic motoneurone firing, again providing the greatest sensitivity to detect a CST volley via its evoked EMG activity. We expect that CST activation will also occur at the same intensity when the subject is at rest, since stimulation is distant to the axon initial segment and hence insensitive to the level of cortical excitability. This is so, even though the weak CST volley produced by a near-threshold PF stimulus is unlikely to generate a measurable MEP in resting motoneurons.

In the second experiment, subjects were at rest. Motor cortex was stimulated either alone, or preceded by PF stimulation (3, 5 or 7 ms intervals; intensities as before). Here, the coil was inverted to generate an upward brain current, previously found to be optimal for MEP suppression (Ugawa et al., 1995). Around 20 stimuli at each intensity were delivered.

EMG and stimulus markers were captured direct to a computer. Off-line analysis separated responses according to condition and compiled averages of rectified EMG. Single sweep responses were measured as the area between the MEP onset and offset, judged from the averaged response. Responses to PF stimulation alone were normalised as a percentage of the mean background level over the 20 ms prior to the stimulus. Significance was assessed by comparing single trial responses with a similar duration of the pre-stimulus background (paired *t*-test). Conditioned responses to motor cortical stimulation were expressed as a percentage of the unconditioned response; significant suppression was assessed with unpaired *t*-tests.

## 3 Results

**Fig. 1:**
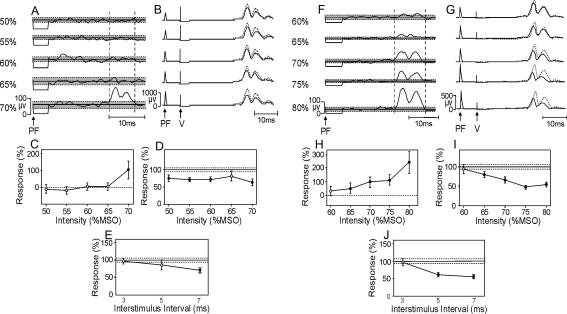
Single subject results. (A) Averaged rectified EMG (1DI) following PF stimulation at different intensities. Vertical dashed lines show response region. Grey shading shows mean baseline ± 2 standard errors. (B) MEPs following motor cortical stimulation (intensity 44% MSO). Dotted lines are unconditioned responses; solid lines show responses conditioned by PF stimulation. (C) Response magnitude versus intensity. (D) Conditioned response magnitude as a percentage of unconditioned responses. (E) Average time course of MEP suppression at DMT-10%. In (C, D and E), error bars show standard errors; horizontal dotted lines in (D and E) show standard error of unconditioned response. Filled circles indicate significant points (*P* < 0.05). (F–I) As in (A)–(D) for a different subject. Motor cortical stimulation in this subject was performed at 36% MSO. (J) Average time course of MEP suppression at DMT-5% for this subject.

Figs. 1A–E show the results from an individual subject, in whom the initial estimate of the threshold for a direct response to PF stimulation was 70% MSO. Fig. 1A shows a clear averaged EMG response at this intensity. No significant direct responses were present at lower PF intensities (Fig. 1C).

Conditioned MEPs (7 ms ISI) are illustrated for this subject in Fig. 1B. Suppression was elicited consistently at all intensities of PF stimulation. Therefore, for this individual, stimulating at intensities of DMT-5% and below seems to generate MEP suppression in the absence of direct CST activation.

Figs. 1F–J show data from a subject whose PF threshold was initially estimated as 80% MSO. Significant suppression was seen as low as 65% MSO (Fig. 1I), however there was also a significant direct response to PF stimulation at this intensity (Fig. 1H). The observed suppression cannot therefore be unambiguously assigned to a cerebellar pathway. Moreover, although the response was not significant at 60%, the response region was above baseline, and the characteristic bifid peak produced by an MEP in rectified EMG was clearly visible (Fig. 1F, 60%). It would be unsafe to conclude that there was no CST activation at an intensity of 60% MSO in this subject.

ISI timecourses for individual subjects (Figs. 1E and J) confirmed that MEPs were suppressed at 5 and 7 ms intervals, but not at 3 ms, consistent with previous reports (Ugawa et al., 1995; Matsunaga et al., 2001). Previous work has used PF stimulus intensity 5–10% MSO below the threshold estimated with the coil in the midline (Ugawa et al., 1994a, 1995; Matsunaga et al., 2001). At this intensity, we found 9/11 subjects had significantly suppressed MEPs. However, all nine also had a direct response in at least one muscle.

Working from the direct response threshold subjectively estimated with the coil at the midline is clearly unsound. Stimuli weaker than this with the coil placed more lateral do produce CST activation, confounding the interpretation of MEP suppression. It is unlikely that this was due to a lower threshold in the lateral position, as a previous study demonstrated that the midline was the optimal position for CST activation (Ugawa et al., 1994b). Alternatively, weak responses following near-threshold stimuli may be missed in single sweeps, causing threshold to be assigned higher than it actually is. Some previous reports determined threshold from averaged MEPs (Daskalakis et al., 2004), but many authors do not clearly describe the method used. Choosing the intensity of PF stimulation more carefully might therefore allow selectivity for cerebellar pathways. In order to test this, we rectified and averaged responses to PF stimuli; threshold was then redefined according to the presence of significant MEPs in these averages. This is a sensitive means of determining threshold; we designated this estimate the objective direct response threshold (ODRT).

**Fig. 2:**
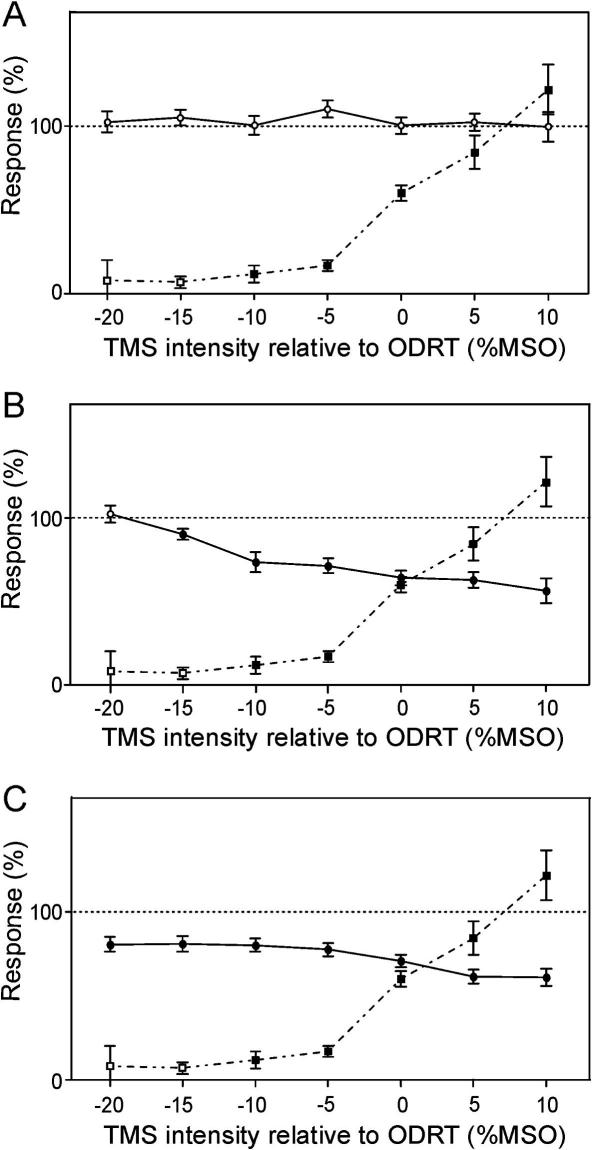
Average direct response and MEP suppression for each ISI is plotted versus stimulus intensity (expressed relative to ODRT). Solid lines plot average MEP suppression at ISI’s of 3 ms (A), 5 ms (B) and 7 ms (C). Filled circles indicate significant points (*P* < 0.05). Dashed lines show average direct response size following PF stimulation for comparison. Filled squares indicate significant points (*P* < 0.05). All curves are averaged over the 11 subjects who participated in this study. Error bars show standard errors.

Averaged data are presented as a function of intensity relative to ODRT in Fig. 2. For the 3 ms interval (Fig. 2A), MEP suppression (circles) was not evident at any intensity. By comparison, a clear effect on motor cortical output was observed with the longer intervals. For 5 ms, significant MEP suppression was generated at 15% MSO below ODRT (Fig. 2B), whilst suppression at the 7 ms ISI was significant at all intensities tested (Fig. 2C). Overlain on the plots of Fig. 2 is the averaged direct response (squares). When we averaged across subjects, small but significant responses were seen even 10% below ODRT. This suggests that even when we determined threshold using a highly objective, sensitive method in single subjects, weak responses were still present at lower intensities. These could only be revealed by the increased statistical power of pooling data across subjects.

**Fig. 3:**
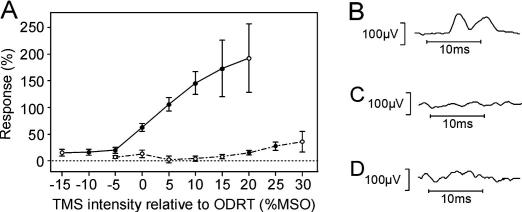
Effect of coil orientation on direct responses. (A) Direct response size as a function of stimulus intensity relative to ODRT, averaged across seven subjects. Solid line indicates measurements with brain current downwards; dashed line indicates brain current upwards. Solid circles indicate responses significantly different from zero (*P* < 0.05). Error bars show standard errors. (B and C) Example response from one subject (1DI muscle) with an intensity of 74%, brain current downwards (B) and upwards (C). (D) Same subject, intensity 84%, brain current upwards.

One important parameter of PF stimulation is the orientation of the TMS coil. It has previously been shown that an orientation yielding an upwards brain current (as used here) generates the greatest MEP suppression. In a subset of seven subjects, we tested the direct motor responses when the coil was oriented either to produce this current direction, or the opposite. Fig. 3A plots the response amplitude averaged across subjects versus intensity relative to ODRT. The two plots indicate responses generated with a brain current oriented downwards (solid line) and upwards (dashed line). Significant responses were generated at 10% below ODRT with the downwards brain current, but only at 20% above ODRT with the upwards brain current. Fig. 3B illustrates the response seen in one subject at 74% of maximal stimulator output with a downward brain current; by contrast there was no response at this level (Fig. 3B) or at a higher intensity (84%; Fig. 3C) with the coil inverted.

## 4 Discussion

Magnetic stimulation of the PF probably has complex actions, and a variety of peripheral and central pathways could contribute to its effects (Meyer et al., 1993). Antidromic action potentials in the CST could plausibly produce MEP suppression, as corticospinal neurones are known to make recurrent collaterals to inhibitory interneurones in the cortex (Renaud and Kelly, 1974). Other brainstem pathways, such as the spinothalamic tract or dorsal columns, could also produce MEP suppression, although examination of their contribution is beyond the scope of this paper. To be certain that MEP suppression is mediated by cerebellar pathways, we must exclude all other possibilities. This requires a particularly stringent standard of evidence: to exclude a CST contribution, for example, it is necessary to show with high confidence that there is *no* activation of corticospinal fibres. In this study, we identified several difficulties in doing this.

Firstly, it is necessary to measure the threshold for a direct response to corticospinal stimulation. When we did this using online observation of single sweep responses, we generally overestimated threshold. Subsequent observation of averaged traces revealed significant averaged responses at intensities below our initial threshold estimate. Hanajima et al. (2007) also reported an important dependence of threshold estimates on the method used. However, even correcting the threshold measure using these averages (ODRT) did not give the required level of confidence that no response was present. When we averaged across subjects, small but significant responses at intensities below ODRT could be revealed (Fig. 2).

Secondly, antidromic activation of corticospinal axons has the greatest potential to generate MEP suppression following PF stimulation (via intra-cortical recurrent collaterals). Such activation cannot be assessed directly; we can only measure the generation of responses in muscle, which results from orthodromic activation of corticospinal axons and subsequent synaptic action on motoneurons. We found a marked difference in the efficacy of upward versus downward orientations of stimulating current in generating muscle responses, which was opposite to that previously reported by Ugawa et al. (1994b). Motivated by this discrepancy, we took care to verify the direction of induced current by observing the potential across a wire placed under the coil; this confirmed that the optimal direction in our subjects was indeed opposite to that found by Ugawa et al. (1994b). This difference may relate to the more lateral location of the coil in our experiments compared to the previous work. The most plausible explanation for the different efficacy of upward versus downward current is the presence of anode block with the upward current. Stimulation over the brainstem, where the tracts are small, may be sufficient to concentrate current and generate a focal hyperpolarisation. This would prevent transmission of MEPs, but suppression via antidromic activation and recurrent inhibition would remain unaffected. We conclude that only if no direct response can be seen following PF stimulation with a downward brain current is it safe to assign MEP suppression seen with an upward brain current to non-CST pathways.

Despite the evidence presented here of CST activation, it is likely that such a large stimulus to the PF will also activate part/all of the cerebellum. It is therefore difficult to identify exactly how much each structure contributes to the effects on MEPs. Lesions of the cerebellum and cerebellar efferent pathways can interfere with MEP suppression, indicating a potential role for a cerebellothalamocortical pathway (Matsunaga et al., 2001; Ugawa et al., 1994a, 1997). Other studies have shown preserved MEP suppression after cerebellar lesion (Meyer et al., 1993, 1994). Ugawa et al. (1994a, 1997) suggested that cerebellar stimulation could be a useful tool to distinguish disease of cerebellar outflow (yielding impaired suppression) from that of cerebellar afferent pathways (suppression normal). However, disruption of the cerebellar outflow via thalamus to cerebral cortex is likely to produce associated changes in cerebral cortical function, which could plausibly include the networks underlying recurrent inhibition from CST activation. Abnormal suppression would then be seen in patients with cerebellar dysfunction, but the test would not directly measure the cerebellar pathology. This distinction becomes of key importance when PF suppression of MEPs is used to investigate an unknown disease. In that case, the confounded cerebellar and CST responses to PF stimulation which we have demonstrated will make unambiguous interpretation of results impossible.

Given the non-selective nature of TMS over the PF, its utility as an experimental tool will depend on the purpose to which it is put. If all that is desired is to activate the cerebellum, this can probably be achieved. However, if coactivation of other structures would confound the results obtained, the method must be used with considerable caution.

Recently, there has been a surge of interest in low frequency repetitive TMS (rTMS). With this technique, it is possible to create ‘virtual lesions’, reversibly inactivating regions of cerebral cortex to investigate their normal function. A range of reports have emerged investigating cerebellar function using rTMS over the PF (Théoret et al., 2001; Del Olmo et al., 2007; Jäncke et al., 2004; Miall and Christensen, 2004). It may be that some of the consequences of PF rTMS do indeed result from disruption of cerebellar pathways. However, the profound recurrent inhibition from repetitive antidromic CST activation would also severely impair motor performance, limiting the conclusions which can be drawn from these studies.

In this report, we have shown that CST activation occurs at intensities routinely used to stimulate over the PF (Fig. 2). Although it remains possible that the cerebellum contributes to the observed suppression at this level, we conclude that confounding activation of other structures will generate ambiguous results. Therefore, a highly conservative approach must be taken if this method is to be used as a specific assessment of cerebellar function. Intensities 15–20% below threshold should be used and suitable controls undertaken in order to verify the absence of activity in other motor pathways. Our results agree with reports that demonstrate some patients with cerebellar defects continue to show motor cortical suppression following PF stimulation (Ugawa et al., 1994a, 1997; Meyer et al., 1993, 1994).
